# *Pseudomonas* in biotechnology: An annotated selection of World Wide Web sites relevant to the topics in *microbial biotechnology*

**DOI:** 10.1111/1751-7915.14081

**Published:** 2022-05-30

**Authors:** Lawrence P. Wackett

**Affiliations:** ^1^ Department of Biochemistry, Molecular Biology & Biophysics BioTechnology Institute, University of Minnesota St. Paul Minnesota 55108 USA

## Protein expression in *Pseudomonas fluorescens*

https://pubmed.ncbi.nlm.nih.gov/21968453/

A *Pseudomonas fluorescens* strain was shown to be highly effective in expressing various proteins at high levels and in a soluble and active form.

## *Pseudomonas* mammalian protein production platform

https://data.epo.org/gpi/EP1692282A1

This page provides links to patents describing *Pseudomonas*‐based mammalian protein production systems designated as the Pfenix system.

## *Pseudomonas putida* for industrial biocatalysis

https://pubmed.ncbi.nlm.nih.gov/29758287/

This review article focuses on the virtues of *Pseudomonas putida* for industrial chemical production.

## *Pseudomonas* and novel metabolic pathways

https://portlandpress.com/essaysbiochem/article/65/2/319/229203/Towards-robust-Pseudomonas-cell-factories-to

While *Pseudomonas* spp. have intrinsically diverse metabolism, this review posits that they are also good platforms for engineering metabolic pathways.

## Engineering robustness in *Pseudomonas* metabolism

https://journals.asm.org/doi/10.1128/mbio.01794‐21

This study investigated the role of polyhydroxyalkanoate formation and mobilization in achieving better control and robustness of *Pseudomonas* metabolism.

## *Pseudomonas putida*: MicrobeWiki

https://microbewiki.kenyon.edu/index.php/Pseudomonas_putida

This page provides good overall information about *Pseudomonas putida*.

## *Pseudomonas* genome database

https://www.pseudomonas.com

Most biotechnological applications will use genomic data and this website provides entries to sequences and tools pertaining to more 9000 *Pseudomonas* strains.

## *Pseudomonas* strain information

https://lpsn.dsmz.de/search?word=Pseudomonas

This site provides information on 590 isolated *Pseudomonas* strains. Information includes isolation origin, and links to related data.

## *Pseudomonas* in BacDive

https://bacdive.dsmz.de/search?search=Pseudomonas

A search of *Pseudomonas* in the BacDive database provides information on strains currently and previously designated as *Pseudomonas* spp.

## *Pseudomonas* Taxonomy

https://www.ncbi.nlm.nih.gov/Taxonomy/Browser/wwwtax.cgi

This site of the National Center for Biotechnological Information contains a very large collection of *Pseudomonas* strains. The majority do not list a species designation.

## Anaerobicity and *Pseudomonas*

https://bmcmicrobiol.biomedcentral.com/articles/10.1186/s12866‐020‐02058‐1

Model pseudomonad like *P. putida* KT2440 grow aerobically. This modeling study indicates that implementation of anerobic respiration in that organism would require extensive sets of additional genes.

## Pseudomonas chlororaphis

https://bmcmicrobiol.biomedcentral.com/articles/10.1186/s12866‐021‐02420‐x

*P. chlororaphis* is being effectively used as a biological control agent in agriculture for preventing fungal diseases of crops.

## Pseudomonas on the Biocatalysis/Biodegradation Database

http://eawag-bbd.ethz.ch/servlets/search

A search of the Biocatalysis/Biodegradation Database with the term “*Pseudomonas*” gives a large set of microorganisms and the biodegradation or biocatalysis pathway for which they have been studied.

## *Pseudomonas* and stress response

https://www.frontiersin.org/articles/10.3389/fmicb.2021.660134/full

*Pseudomonas* strains show diverse capacity to deal with stresses through making protective substances, enlisting chaperones, altering membranes and resisting solvents. These properties can make them more robust for biotechnological applications.

